# Short- and long-term scaling behavior of blood pressure and pulse arrival time during sleep in healthy controls and patients with obstructive sleep apnea

**DOI:** 10.1371/journal.pone.0339755

**Published:** 2026-07-01

**Authors:** Karsten Berg, Jan W. Kantelhardt, Martin Glos, Thomas Penzel, Niels Wessel, Ronny P. Bartsch

**Affiliations:** 1 Department of Physics, Humboldt-Universität zu Berlin, Berlin, Germany; 2 Institute of Physics, Martin-Luther-Universität Halle-Wittenberg, Halle, Germany; 3 Center for Sleep Medicine, Charité-Universitätsmedizin Berlin, Berlin, Germany; 4 Department of Human Medicine, MSB Medical School Berlin GmbH, Berlin, Germany; 5 Department of Physics, Bar-Ilan University, Ramat-Gan, Israel; Coventry University, UNITED KINGDOM OF GREAT BRITAIN AND NORTHERN IRELAND

## Abstract

Obstructive sleep apnea (OSA) is characterized by recurrent respiratory events that trigger autonomic arousals and blood pressure (BP) surges, contributing to elevated cardiovascular risk. Photoplethysmography (PPG)-derived timing markers such as pulse arrival time (PAT) are frequently used as noninvasive surrogates of BP dynamics, yet their interpretation is confounded by the pre-ejection period and peripheral vascular effects. Here, we used detrended fluctuation analysis (DFA) to quantify short- and long-term scaling exponents of continuous blood pressure (Portapres), PPG-, and PAT-derived signals across sleep stages in healthy individuals and patients with OSA. Directly measured systolic and diastolic BP exhibited a robust short- to long-term crossover across all sleep stages, with elevated short-range exponents ($\alpha_{1}>1$) and lower long-range exponents ($\alpha_{2}<1$), reflecting well-organized autonomic and vascular control. In OSA, this crossover persisted but was visibly attenuated, consistent with reduced short-term adaptability of cardiovascular regulation. In contrast, PAT-based indices showed substantially weaker short-range correlations and minimal crossover structure. Systolic PAT displayed almost no separation between $\alpha_{1}$ and $\alpha_{2}$, and PPG-derived measures exhibited scaling patterns that differed fundamentally from BP. Across modalities, PAT (whether derived from BP or PPG) failed to reproduce the multiscale organization characteristic of beat-to-beat BP dynamics. Group comparisons further identified systolic BP scaling, particularly the short-range exponent $\alpha_{1}$, as the most sensitive marker of cardiovascular dysregulation in OSA, whereas PAT and PPG provided complementary but physiologically distinct information related to peripheral vascular and autonomic modulation. These findings demonstrate that PAT and PPG timing measures should not be used as surrogates for BP in fractal or scaling analyses and underscore the unique diagnostic value of BP-derived scaling behavior for assessing cardiovascular regulation during sleep.

## 1. Introduction

Obstructive sleep apnea (OSA) is a severe sleep disorder characterized by repeated upper-airway collapses during sleep that lead to hypoxaemia, frequent arousals, and fragmented sleep architecture [1,2]. It is highly prevalent, with recent estimates suggesting that nearly one billion people are affected globally [3]. Moreover, OSA is associated with hypertension, coronary disease, stroke, and increased mortality, making accurate detection and staging essential for clinical management [4]. Modern screening approaches increasingly rely on non-invasive physiological signals that are easier to deploy than full polysomnography (PSG) [5].

Fluctuations in blood pressure (BP) play a central role in the pathophysiology of OSA: repeated apnea events trigger sympathetic surges, transient BP elevations, and altered baroreflex sensitivity, linking OSA severity directly to cardiovascular risk [6,7]. For cardiac interbeat interval time series, earlier work has demonstrated that the scale-invariant organization is altered in OSA [8]. Therefore, continuous assessment of BP dynamics may provide complementary insight into autonomic cardiovascular regulation during sleep.

Photoplethysmography (PPG) has emerged as a promising tool for sleep monitoring and OSA detection [9]. Timing parameters derived from PPG and ECG, such as pulse arrival time (PAT) and pulse transit time (PTT), are particularly useful because they reflect aspects of cardiovascular regulation and autonomic control. PTT, defined as the time required for a pulse wave to travel between two arterial sites, inversely relates to arterial stiffness and BP [10]. PAT additionally includes the pre-ejection period (PEP) of the heart and therefore incorporates cardiac contractility and sympathetic activation [11,12]. Since apnea episodes elicit characteristic autonomic and hemodynamic responses, both PAT and PTT exhibit measurable fluctuations during sleep-disordered breathing [13–15].

Beyond discrete event detection, the temporal organization of cardiovascular signals provides additional insight into physiological regulation [16]. Detrended fluctuation analysis (DFA) quantifies the scaling of signal variability across different time ranges and yields short- and long-term scaling exponents ($\alpha_{1}$, $\alpha_{2}$) that relate to autonomic control mechanisms [17,18]. Alterations in these exponents have been associated with impaired adaptability of cardiovascular and respiratory systems across sleep stages, circadian phase, and disease states [19–23]. Recent developments in Network Physiology have expanded this perspective, showing how multiscale coupling, redundancy, and synergy shape cardiovascular dynamics under sleep and postural challenge [16,24]. Additional work has demonstrated that physiological variability is best understood within a framework of constrained disorder, where deviations from healthy multiscale structure reflect impaired flexibility of autonomic and vascular control [25]. Together with advances in analyzing cardio-respiratory and vascular interaction gradients [24,26], these insights highlight the need to evaluate cardiovascular signals not only through their average properties but through their multiscale organization.

Following this multiscale/multi-organ approach, in a recent study, we examined DFA scaling behavior across several physiological systems, including beat-to-beat intervals, respiratory intervals, EEG amplitudes, and pulse transit times, and observed a consistent crossover from short-term to long-term correlations in most signals, except for PTT [17]. This absence of a clear $\alpha_{1}-\alpha_{2}$ crossover is in contrast with earlier findings for direct BP measurements [27], and calls into question how well PAT/PTT serves as a surrogate for BP dynamics across time scales.

To address this, we analyzed data from the previous EU project DAPHNet [28], which uniquely combines full PSG and simultaneously recorded continuous non-invasive BP measurements. This configuration allows direct comparison of PAT and PPG-derived pulse dynamics with beat-to-beat BP fluctuations across sleep stages, and enables a systematic evaluation of whether PAT captures the multiscale temporal structure of BP in both healthy subjects and individuals with OSA.

## 2. Materials and methods

### Dataset and study protocol

The study protocol and measurement setup of the DAPHNet project (2006–2009) have been described in detail elsewhere [28]. The original DAPHNet study was approved by the Ethics Committee of Charité-Universitätsmedizin Berlin (approval number EA1/034/07), and all participants provided written informed consent. The original investigation focused on autonomic and cardiovascular responses to arousals in obstructive sleep apnea (OSA), using healthy subjects as controls. The analyses reported in the present manuscript constitute a secondary analysis conducted under the original ethics approval. For this secondary analysis, fully anonymized recordings were accessed in July 2022 for research purposes. The authors had no access to any personal identifiers at any point during data handling or analysis.

Participants underwent two consecutive nights of full in-lab PSG (Embla Inc., Broomfield, CO). Recordings included EEG (C3/A2, C4/A1), bilateral EOG, chin and tibialis EMG, ECG, airflow, respiratory effort, SpO_2_, and finger photoplethysmography (PPG). Continuous non-invasive beat-to-beat blood pressure (BP) was recorded using a Portapres Model 2 device (Finapres Medical Systems), which alternates finger cuffs and applies hydrostatic height correction based on the volume-clamp method [29]. Night 1 served as adaptation; night 2 was manually scored according to AASM criteria. Of the original 44 subjects, 40 recordings were available for re-analysis: two were unavailable and two OSA subjects were excluded due to missing BP signals. Group-level descriptive statistics are provided in Table 1.

**Table 1:** Demographic and clinical characteristics of OSA and control groups. BMI-Body Mass Index; AHI-Apnea–Hypopnea Index; RERA-Respiratory Effort–Related Arousal.

	OSA	Controls
Number of subjects	17	23
Hypertension, n (%)	7 (41%)	1 (4%)
Age, range (years)	36–76	28–69
Age, mean ± SD (years)	54.5 ± 11.1	51.1 ± 8.8
BMI, mean ± SD (kg/m^2^)	30.1 ± 5.2	24.5 ± 2.9
Sex, female/male	8/9	12/11
Antihypertensive medication, n (%)	7 (41%)	1 (4%)
AHI, mean ± SD (events/h)	19.9 ± 15.6	1.4 ± 1.1
Arousals, mean ± SD	101.9 ± 80.0	47.0 ± 23.7
Total arousals, n	1,733	1,082
Respiratory arousals, n (%)	1,264 (73%)	68 (4%)
Limb‐movement arousals, n (%)	165 (10%)	307 (18%)
Spontaneous arousals, n (%)	222 (13%)	668 (39%)
RERA, n (%)	82 (5%)	39 (2%)

### Subjects

Controls were age- and sex-matched to OSA subjects. OSA was defined by an apnea–hypopnea index (AHI) >5 events/h (controls: AHI < 5 events/h). Exclusion criteria followed [28] and included age < 18 years, BMI > 40 kg/m^2^, substance abuse, thyroid dysfunction, chronic pain, neurological or psychiatric disorders, significant cardiovascular or pulmonary disease, periodic limb movement index (PLMI) >5 events/h, and recent clinical trial participation or intercontinental travel.

### Physiological signals

We analyzed the following signals from night 2:

- ECG (200 Hz),
- PPG via pulse oximetry (100 Hz),
- continuous beat-to-beat BP from the Portapres system (200 Hz).

Signals were synchronized using internal device timestamps, compensating for known Embla system delays. Preprocessing steps included baseline removal, band-pass filtering and artefact suppression (full filter specifications in Supplementary Material).

### Morphologic features

**Fig 1:**
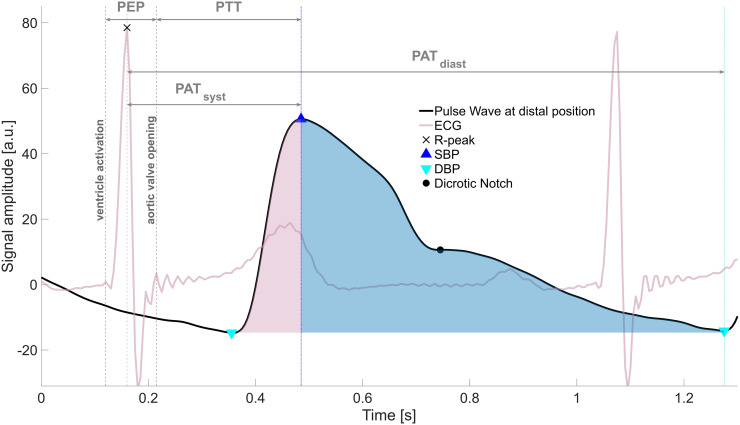
Characteristic features of the arterial blood pressure pulse wave measured at the finger tip. The red and blue shaded regions represent the anacrotic (rising) and catacrotic (falling) phases of the pulse wave, extending from the diastolic minimum (DBP) to the systolic maximum (SBP) and back. The first local minimum on the falling limb, the dicrotic notch, reflects aortic valve closure and marks the transition between systolic and diastolic components of the waveform. Pulse arrival time (PAT) is defined as the interval from a fiducial point within the QRS complex (typically the R-peak) to the arrival of the peripheral pulse waveform at its systolic or diastolic feature. PAT includes the pre-ejection period (PEP) and thus differs from pulse transit time (PTT), which reflects only the vascular propagation delay and requires identification of aortic valve opening or two peripheral PPG sensors for accurate estimation [11,12]. Fiducial points are aligned with waveform peaks in this schematic for illustrative clarity. In our analysis, PAT_sys_ was defined as the interval from the R-peak to the SBP maximum in BP signals and to the systolic peak in PPG signals. PAT_dia_ was defined as the interval from the R-peak to the diastolic minimum (DBP in BP signals; first diastolic minimum following the systolic peak in PPG signals). The dicrotic notch was not used as a fiducial marker.

Fig 1 illustrates systolic and diastolic features of the BP pulse (as observed at the finger tip) and their relation to PPG morphology. Systolic BP (SBP) was defined as the pulse maximum; diastolic BP (DBP) as the subsequent local minimum on the falling limb.

Pulse arrival times (PAT) were defined as:

- **PAT**_**sys**_: ECG R-peak to SBP (Portapres),
- **PAT**_**dia**_: ECG R-peak to DBP (Portapres).

The dicrotic notch was not used as a fiducial point. As PAT includes the pre-ejection period (PEP), it is physiologically distinct from pulse transit time (PTT), which requires two arterial measurement sites and reflects purely vascular propagation delay [11,12]. For PPG signals, PAT_sys_ and PAT_dia_ were defined analogously as the intervals from the R-peak to the PPG systolic peak and to the first PPG minimum following that peak, respectively.

### Data preprocessing

All analyses were performed in MATLAB (R2023a). ECG signals were corrected for baseline drift using cascaded median filters, and R-peaks were detected using the Benitez algorithm [30]. PPG and BP signals were filtered with zero-phase Butterworth filters to suppress high-frequency noise while preserving pulse morphology. Detailed filter orders, cutoffs, and resampling steps are provided in the Supplementary Material.

### Beat segmentation and validation

**Fig 2:**
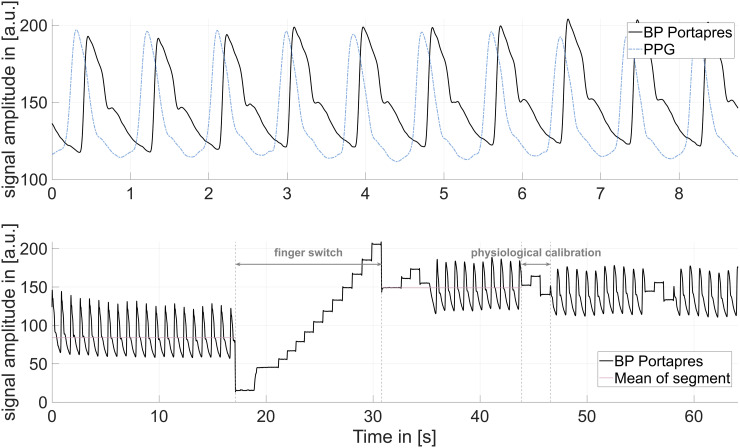
Typical artifacts in Portapres (BP) and PPG (pulse oximetry) recordings. *Top:* Example of simultaneous BP (solid line) and PPG (blue dashed line) waveforms. The PPG pulse wave lacks a distinct dicrotic notch and shows a temporal delay relative to the BP waveform. This absence of the dicrotic feature is likely attributable to age-related vascular stiffening, which attenuates the reflected wave component and highlights the physiological and morphological differences between the two signals. Whereas BP primarily reflects cardiac function and central arterial dynamics, the PPG pulse wave is more strongly influenced by peripheral vascular properties. *Bottom:* Representative artifacts in the Portapres BP signal. The device alternates measurement fingers approximately every 30 minutes to prevent vasoconstriction, producing abrupt baseline offsets. In addition, internal physiological recalibrations occur every 10–70 beats depending on signal quality [32,33]. Both finger-switching and calibration events, as well as brief signal losses, lead to missing or invalid beats. In the present analysis, such segments were excluded, and only relative beat-to-beat changes in systolic and diastolic pressure (SBP and DBP) were evaluated, as these contain physiologically meaningful variability [34].

Beats were segmented using consecutive ECG R-peaks as boundaries. BP and PPG beats were aligned to the same R-peaks to ensure modality synchronization. Beat morphology was assessed by correlating each beat with a subject-specific template; beats failing modality-specific correlation thresholds were excluded. SBP and DBP were identified using peak-valley detection refined within a ±0.2 s window around template extrema. Beats with physiologically implausible values (e.g., SBP > 200 mmHg, DBP < 10 mmHg) were discarded. RRIs, PATs, and BP values violating established physiological limits [17,31] were excluded. Segments with missing signal or with calibration artifacts were also ignored (Fig 2). Only uninterrupted sequences of ≥20 validated beats were used for further analysis.

### Sleep staging

Sleep stages were scored manually according to AASM rules. Because N1 episodes were sparse and typically too short for DFA, analyses focused on wake, N2, N3, and REM sleep.

### Detrended fluctuation analysis

Detrended fluctuation analysis (DFA) [35,36] quantifies correlation properties of nonstationary time series. DFA2 (quadratic detrending) was applied to nine beat-to-beat time series: ECG-derived RRI; Portapres-derived SBP, DBP, PAT_sys_, PAT_dia_; and PPG-derived systolic peak, diastolic minimum, PAT_sys_, and PAT_dia_. Short- and long-term scaling exponents were defined as:


$$\begin{array}{c} \alpha_{1}:\;s=6\text{-}16\text{ beats},\;\alpha_{2}:\;s=50\text{-}200\text{ beats}. \end{array}$$

**Fig 3:**
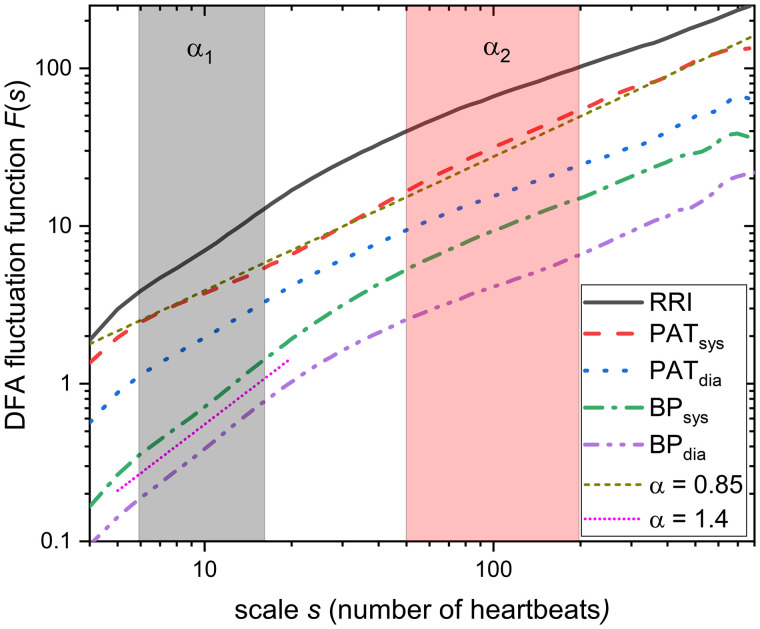
DFA fluctuation functions for cardiovascular time series. Double-logarithmic plots of the average DFA fluctuation function *F*(*s*) versus time scale *s* for 23 healthy subjects. Shown are the results for ECG-derived RR intervals (RRI), systolic and diastolic BP values from the Portapres device, and the corresponding systolic and diastolic pulse arrival times (PAT_sys_, PAT_dia_). BP-derived signals display strong short-term correlations (typical $\alpha_{1}\approx 1.4$) for both systolic and diastolic values, similar to the behavior observed in RRI. In contrast, PAT signals exhibit markedly weaker short-term correlations, and the crossover between short- and long-term scaling regimes is substantially reduced or absent, particularly for systolic PAT. Grey and red shaded regions indicate the fitting windows used to estimate the short-term ($\alpha_{1}$, 6-16 beats) and long-term ($\alpha_{2}$, 50-200 beats) scaling exponents, respectively. Straight reference lines with slopes α=0.85 and α=1.4 are shown as guides to the eye.

Window sizes were defined in beats rather than seconds because beat-to-beat intervals constitute the natural physiological time base for cardiovascular variability: baroreflex regulation, autonomic modulation, and pulse-wave propagation all operate on a per-beat basis. Resampling to equal time intervals requires interpolation across irregularly spaced events and would convolve heart-rate variability with the variability of the analyzed signal, complicating direct comparison across RRI, BP, and PAT. The use of beat-based windows (6–16 and 50–200 beats) is also consistent with prior DFA studies of cardiovascular dynamics during sleep [17,31], allowing direct comparison with the existing literature. Only scaling fits with *r*^2^ > 0.9 were accepted (Fig 3).

### Statistical analysis

Because $\alpha_{1}$ and $\alpha_{2}$ were not normally distributed (Shapiro-Wilk, *p* < 0.05), non-parametric methods were used. The primary analyses focused on differences in scaling behavior across the physiological measures. Within each subject group (healthy, OSA), Kruskal-Wallis tests were applied across the five Portapres-derived measures (RRI, SBP, DBP, PAT_sys_, PAT_dia_) and across the four PPG-derived measures (systolic peak, diastolic minimum, PAT_sys_, PAT_dia_) for each sleep stage. Significant effects were examined using Bonferroni-corrected pairwise Wilcoxon tests. Stage-related differences within each physiological measure were assessed using Kruskal-Wallis tests across wake, REM sleep, N2, and N3, followed by Bonferroni-corrected pairwise comparisons where appropriate. Between-group effects (healthy vs. OSA) were evaluated separately for each physiological measure and sleep stage using Kruskal-Wallis and Wilcoxon tests. All analyses were performed in R (packages rstatix and tidyverse). Detailed test statistics and *p*-values for within-group stage effects, pairwise stage comparisons, and between-group differences are provided in S1–S3 Tables.

## 3. Results

DFA (Fig 3) revealed distinct short- and long-term scaling behaviors across cardiovascular signals. Blood pressure (BP) recordings obtained with the Portapres system showed strong short-term correlations, with $\alpha_{1}\approx 1.4$ for both systolic and diastolic components, consistent with the behavior of RR intervals. A clear crossover from $\alpha_{1}>1$ to $\alpha_{2}<1$ was present, indicating strong local coupling and reduced short-term variability. In contrast, pulse arrival times (PAT) exhibited markedly lower short-term exponents and relatively stronger long-term correlations, resulting in a substantially reduced or absent crossover. This pattern suggests that PAT reflects a different temporal organization from pressure-derived measures, dominated by peripheral vascular influences rather than central arterial dynamics.

**Fig 4:**
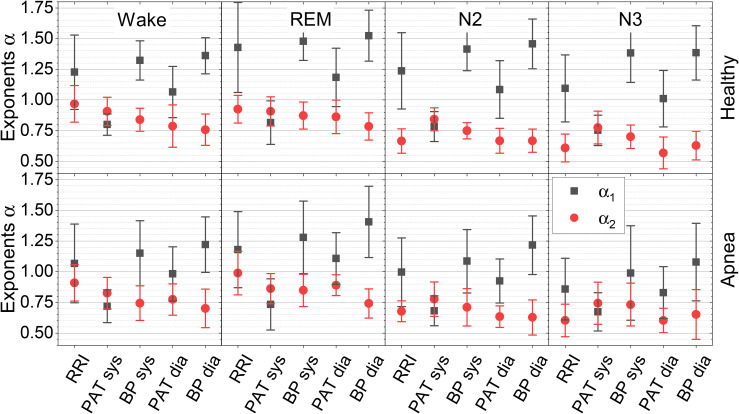
Short- and long-term scaling exponents for Portapres-derived measures in healthy and OSA subjects across sleep stages. Average short-term ($\alpha_{1}$, black) and long-term ($\alpha_{2}$, red) DFA exponents for RR intervals (RRI), systolic PAT (PAT_sys_), systolic BP (SBP), diastolic PAT (PAT_dia_), and diastolic BP (DBP) during wake, REM, light sleep (N2), and deep sleep (N3), shown separately for healthy subjects (top panel) and patients with OSA (bottom). Error bars denote standard deviations. In healthy subjects, RRI and systolic and diastolic BP exhibited a pronounced separation between $\alpha_{1}$ and $\alpha_{2}$ across sleep stages, indicating a robust short- to long-term crossover, whereas PAT signals showed only weak or absent $\alpha_{1}-\alpha_{2}$ separation. In OSA, the $\alpha_{1}-\alpha_{2}$ separation for RRI and BP was visibly attenuated relative to healthy subjects, consistent with dampened short-term cardiovascular coupling under sleep-disordered breathing, while PAT_dia_ showed a separation comparable to that of RRI in several stages. Overall, BP-derived measures retained the clearest crossover structure in both groups, whereas PAT-based indices reflected a distinct and more variable temporal organization.

In healthy subjects, this contrast was preserved across all sleep stages (Fig 4, top panel): RR intervals and both systolic and diastolic BP consistently exhibited a clear short- to long-term crossover, with $\alpha_{1}>1$ and $\alpha_{2}<1$. This pattern reflects strong short-term cardiovascular coupling and reduced long-term persistence. In contrast, PAT_sys_ and PAT_dia_ showed only weak or absent separation between $\alpha_{1}$ and $\alpha_{2}$, with the diastolic PAT exhibiting a modest stage dependence only in deep sleep. These results confirm that, in healthy subjects, PAT captures substantially less short-term correlation structure than directly measured BP, consistent with the distinct temporal organization of PTT/PAT reported in earlier work [17].

Short- and long-term scaling patterns in the OSA group are shown in Fig 4 (bottom panel). As in healthy subjects, systolic and diastolic BP displayed a clear separation between $\alpha_{1}$ and $\alpha_{2}$ across sleep stages, while PAT signals again showed only weak or absent crossover. However, the magnitude of the $\alpha_{1}-\alpha_{2}$ difference was slightly reduced in OSA, particularly for RRI and for both systolic and diastolic BP during N2 and N3 sleep, consistent with a dampening of short-term cardiovascular coupling in the presence of sleep-disordered breathing.

Direct comparison of the two groups confirmed these patterns. Systolic BP exhibited the most robust group differences, with consistently larger $\alpha_{1}$ and lower $\alpha_{2}$ in healthy subjects across sleep stages. Diastolic BP showed a similar trend, though the $\alpha_{1}-\alpha_{2}$ separation was most pronounced in N3 sleep. PAT measures provide the weakest differentiation between healthy and OSA subjects: systolic PAT displayed minimal separation of $\alpha_{1}$ and $\alpha_{2}$ in both groups, while diastolic PAT showed modest group differences only in deep sleep.

**Fig 5:**
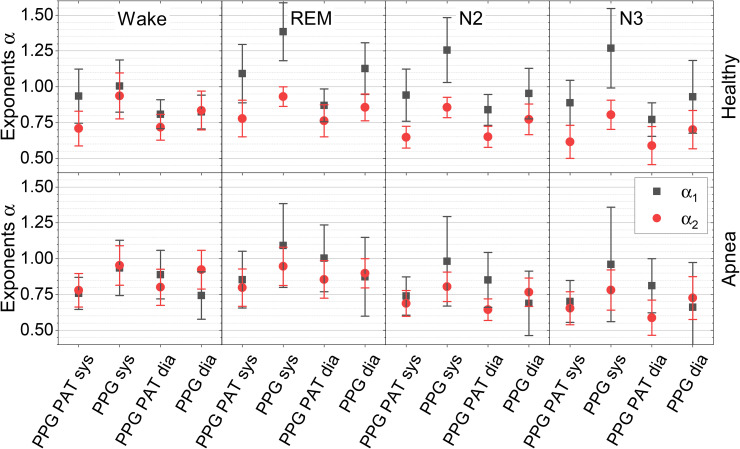
Short- and long-term scaling exponents for PPG-derived measures in healthy and OSA subjects. Average short-term ($\alpha_{1}$, black) and long-term ($\alpha_{2}$, red) DFA exponents for systolic PPG (PPG_sys_), diastolic PPG (PPG_dia_), systolic PAT (PPG-PAT_sys_), and diastolic PAT (PPG-PAT_dia_). Healthy participants (top panel) showed a distinct $\alpha_{1}-\alpha_{2}$ crossover for PPG_sys_ and, to a lesser extent, PPG_dia_, whereas PAT-based indices exhibited much weaker separation. In OSA patients (bottom panel), the $\alpha_{1}-\alpha_{2}$ crossover was substantially reduced or absent across all PPG-derived signals, indicating a loss of multiscale organization of peripheral pulse dynamics in the presence of sleep-disordered breathing. Error bars denote standard deviations.

Fig 5 depicts the corresponding scaling behavior for the PPG-derived signals. In healthy subjects during sleep, systolic PPG (PPG_sys_) exhibited a clear short- to long-term crossover ($\alpha_{1}>\alpha_{2}$), with diastolic PPG showing a similar but slightly weaker pattern. In contrast, PPG-derived PAT measures displayed only modest or absent separation between $\alpha_{1}$ and $\alpha_{2}$, mirroring the reduced crossover observed for ECG-anchored PAT in the Portapres signals.

In OSA patients, the temporal organization of the peripheral pulse wave was markedly changed: the $\alpha_{1}-\alpha_{2}$ crossover evident in healthy subjects was greatly diminished for both systolic and diastolic PPG, indicating a breakdown of short-term correlation structure in the presence of recurrent apneic events. PAT measures remained weakly organized in both groups, with only minor stage-dependent variations.

Together, the PPG results corroborate the Portapres findings and reinforce that PAT, whether derived from BP or PPG, does not reproduce the characteristic scaling properties of beat-to-beat BP fluctuations.

All qualitative patterns presented in this section were confirmed by non-parametric statistical testing; the full set of stage-wise and between-group comparisons is reported in the S1–S3 Tables.

## 4. Discussion

In this study, we examined the multiscale organization of cardiovascular dynamics across sleep stages by comparing DFA scaling exponents of BP-, PPG-, and PAT-derived measures in healthy individuals and patients with OSA. Our analysis addressed the central question raised by earlier work [17]: whether PAT/PTT-type timing measures reproduce the characteristic scaling structure of beat-to-beat blood pressure fluctuations. The present results demonstrate that they do not. Across both modalities (Portapres and PPG) and subject groups (healthy vs. OSA), only direct blood pressure signals as measured by Portapres exhibited a robust and physiologically meaningful short- to long-term crossover. PAT-based indices showed substantially weaker short-term correlations and reduced stage-dependent structure, highlighting their limited utility as surrogates for BP in fractal or scaling-based analyses.

### PAT does not replicate BP scaling: Signal- and group-level interpretation

Across all sleep stages, both systolic and diastolic blood pressure (BP_sys_ and BP_dia_) consistently showed a clear separation between short-term ($\alpha_{1}$) and long-term ($\alpha_{2}$) scaling exponents. This pronounced crossover reflects well-organized cardiovascular regulation, in which baroreflex activity and vascular resistance tightly constrain short-term variability, while slower fluctuations reflect more independent autonomic and hemodynamic influences. Although the crossover was slightly more pronounced in non-REM sleep (N2 and N3), the overall magnitude of the $\alpha_{1}-\alpha_{2}$ difference was comparable for systolic and diastolic BP across all stages. This indicates that both systolic and diastolic pressure dynamics preserve the expected multiscale structure of beat-to-beat blood pressure regulation.

By contrast, the two PAT measures exhibited distinct behaviors. PAT_sys_ showed virtually no separation between $\alpha_{1}$ and $\alpha_{2}$ in any sleep stage, indicating an absence of meaningful short-term correlation structure. PAT_dia_ displayed a slightly clearer crossover, but this effect was modest and largely restricted to non-REM sleep. These findings mirror earlier observations [17] and highlight the mixed cardiovascular nature of PAT: because it includes part of the pre-ejection period (PEP), its short-term dynamics are influenced not only by vascular pulse-wave propagation, but also by cardiac contractility, autonomic modulation, and respiratory effects [12,37]. As a result, PAT exhibits weaker and more variable short-term correlations than directly measured blood pressure, limiting its ability to reproduce the robust $\alpha_{1}-\alpha_{2}$ crossover characteristic of pressure-derived measures.

Between-group comparisons reinforced these differences. Patients with OSA exhibited a flattened scaling profile, with reduced $\alpha_{1}$ and a diminished short- to long-term crossover, particularly for BP_sys_. This pattern suggests impaired short-term autonomic adaptability and reduced baroreflex responsiveness during sleep. Diastolic BP showed similar but slightly weaker tendencies, whereas PAT-based indices again revealed only modest group differences. Overall, BP-derived scaling exponents remained the most effective discriminators between healthy and OSA subjects, while PAT indices showed limited diagnostic sensitivity.

PPG-derived measures displayed fundamentally different behavior from BP. In healthy subjects during sleep, PPG_sys_ exhibited a clear $\alpha_{1}-\alpha_{2}$ crossover, although weaker than that observed for pressure-derived signals. This partial retention of short-term structure indicates some correspondence to BP dynamics, likely mediated by shared autonomic inputs. In OSA, however, this crossover was largely absent, reflecting a loss of short-term organization in peripheral blood-volume dynamics. PAT measures derived from PPG showed the weakest and most inconsistent scaling structure, mirroring the behavior of ECG-referenced PAT derived from the Portapres signal.

Overall, these results demonstrate that PAT, regardless of whether it is derived from BP or PPG, does not reproduce the multiscale organization characteristic of beat-to-beat blood pressure fluctuations. PPG and PAT provide complementary information about peripheral vascular and autonomic function, but neither should be considered a surrogate for BP in fractal or scaling-based analyses.

### Measurement considerations

Continuous blood pressure in the DAPHNet dataset was recorded with the Portapres system, which inherently introduces characteristic calibration artifacts (Fig 2). To prevent finger vasoconstriction, the device alternates the measured finger approximately every 30 minutes, often producing abrupt baseline offsets. In addition, the system performs periodic physiological calibrations every 10–70 beats, depending on signal quality, and occasional signal dropouts may occur [32,33]. All affected segments were removed during preprocessing.

Because calibration shifts and finger switching disrupt absolute pressure baselines, only relative beat-to-beat variability of systolic and diastolic pressure could be analyzed reliably. While this precludes interpretation of absolute BP levels, the remaining variability is physiologically meaningful: fluctuations in SBP and DBP provide insight into autonomic regulation and have been shown to reflect cardiovascular dysregulation in OSA [34]. Thus, although constrained by device-specific limitations, the present analysis captures the temporal organization of pressure dynamics relevant to autonomic and vascular control.

### Device differences

The present study used finger PPG, whereas our earlier work [17] used wrist PPG. Finger and wrist waveforms differ substantially: finger PPG shows higher amplitude and clearer morphology, whereas wrist PPG exhibits weaker correlations with BP and greater motion sensitivity [38]. Wrist-worn devices have also been shown to slightly overestimate systolic and underestimate diastolic pressure, in part due to vertical displacement relative to heart level [39]. These differences underscore the importance of sensor location when interpreting PPG-derived timing indices.

### Physiological interpretation

Several physiological mechanisms explain why PAT differs fundamentally from BP in its scaling behavior. Many earlier studies referred to “PTT” while in fact analyzing ECG‐ referenced PAT (PTT^*^), a distinction that is essential because the pre‐ejection period (PEP) contributes substantially to PAT and introduces strong autonomic and cardiac influences [12,37]. Geddes et al. [40] showed that PAT decreases with rising diastolic pressure, but with weaker correlation than true PTT, particularly at distal measurement sites. Payne et al. [37] further demonstrated that PEP can account for up to one-third of PAT variability, illustrating why PAT is an unreliable proxy for arterial stiffness or instantaneous blood pressure, and why its short-term fluctuations do not mirror those of peripheral pressure.

The PPG waveform additionally reflects local vascular compliance, wave-reflection properties, and sympathetic modulation [41]. The dicrotic notch, first characterized by Dillon [42], attenuates with arterial stiffening, altering waveform shape and thereby influencing scaling behavior. Respiratory and autonomic oscillations impose further low-frequency correlations in the waveform [43–45]. Vascular reflection parameters and stiffness indices also vary with smooth muscle tone, posture, and vasoactive modulation [46,47]. Chen et al. [48] showed that arterial elasticity introduces variability in pulse-wave velocity that is independent of blood pressure, complicating interpretation of PTT/PAT-based estimates.

PAT itself is sensitive to autonomic modulation and posture. Recent work demonstrated that its short-term scaling exponent increases in the supine compared to the sitting position, reflecting greater autonomic stability when lying down [18]. This reinforces that PAT scaling reflects a combined influence of autonomic tone, cardiac electromechanical delay, and vascular propagation, not blood pressure alone.

Taken together, these mechanisms explain why PAT lacks the robust short-term scaling structure observed in BP and why PPG-derived indices differ fundamentally from pressure-derived measures. The observed scaling patterns reflect the interplay of cardiac delay, vascular propagation dynamics, and peripheral autonomic modulation, and therefore should not be interpreted as direct indicators of arterial stiffness or beat-to-beat blood pressure regulation.

### Limitations and future directions

Several limitations of the present study should be acknowledged. First, the sample size (17 OSA patients and 23 healthy controls) is relatively modest, particularly for between-group comparisons. Although our non-parametric statistical framework is appropriate for this setting, larger cohorts will be needed to confirm the robustness of the observed group differences and to detect more subtle stage- and signal-specific effects. The DAPHNet dataset was originally designed for detailed within-subject analyses rather than for large-scale group comparisons, which constrains the statistical power available for the present secondary analysis.

A second limitation is that the OSA group exhibited considerable internal clinical heterogeneity. Apnea-hypopnea index values ranged from mild to severe apnea (AHI = 19.9±15.6 events/h), and 41% of OSA participants received antihypertensive medication (see Table 1), which may itself modulate baroreflex function and short-term cardiovascular variability. BMI also varied substantially across patients. The present sample size did not permit stratified subgroup analyses by OSA severity, comorbidity, or medication status, and group-level results should therefore be interpreted as reflecting the average behavior of a clinically diverse OSA cohort. Future studies in larger and more homogeneous samples (or with sufficient power for severity-stratified analyses) are needed to disentangle the contributions of disease severity, treatment effects, and comorbid hypertension to the observed scaling differences.

Beyond these methodological limitations, several directions for future work emerge from our findings. First, the demonstration that PAT and PPG-derived timing measures do not reproduce the multiscale organization of beat-to-beat BP has direct implications for the growing use of wearable and contact-free cardiovascular monitoring in sleep medicine. Devices that rely on PAT or PPG alone may detect average autonomic changes but are unlikely to capture the short- to long-term scaling structure that distinguishes healthy from pathological cardiovascular regulation. Combining PPG- or PAT-based recordings with intermittent calibrated BP measurements may provide a practical compromise for ambulatory monitoring [10,49]. Second, severity-stratified analyses in larger OSA cohorts could clarify whether scaling-based markers, particularly the short-range exponent $\alpha_{1}$ of systolic BP, track disease severity sensitively enough to support risk stratification or treatment monitoring (e.g., response to CPAP therapy). Third, integrating BP-, PPG-, and PAT-derived scaling measures with respiratory and EEG-based metrics within a Network Physiology framework [16,24] may yield more comprehensive multiscale signatures of cardiovascular dysregulation in sleep-disordered breathing. Together, these directions could help translate scaling-based analyses from research tools into clinically actionable indicators of cardiovascular risk in OSA.

### Conclusion

This study examined the multiscale organization of cardiovascular dynamics during sleep by comparing DFA-derived scaling exponents from continuous blood pressure (BP), PPG, and PAT signals in healthy individuals and patients with obstructive sleep apnea (OSA). Among all modalities, systolic and diastolic BP exhibited the clearest and most consistent separation between short- and long-term scaling exponents, reflecting robust autonomic and vascular regulation in healthy sleep and its attenuation in OSA. These findings demonstrate that beat-to-beat BP dynamics preserve a physiologically meaningful crossover structure that is highly sensitive to sleep-disordered breathing.

In contrast, PPG-derived signals showed a fundamentally different scaling organization. Although systolic PPG retained a partial $\alpha_{1}-\alpha_{2}$ crossover in healthy subjects, this structure was markedly weaker than in BP and largely absent in OSA, underscoring that PPG reflects peripheral blood-volume dynamics rather than central pressure regulation. PAT-based indices, whether derived from BP or from PPG, displayed the weakest and most inconsistent short-term correlations, largely due to their dependence on the pre-ejection period and other cardiac influences. As a result, PAT does not replicate BP scaling and should not be used as a surrogate for blood pressure in fractal or multiscale analyses.

In summary, our results highlight BP scaling as a sensitive marker of cardiovascular dysregulation in OSA, while PPG and PAT provide complementary but physiologically distinct information related to vascular tone, autonomic modulation, and cardiac electromechanical delay. Future work should explore multiscale coupling across combined cardiovascular and respiratory signals and evaluate whether integrating BP-, PPG-, and PAT-based metrics can improve non-invasive risk stratification and treatment monitoring in sleep-disordered breathing.

## Supporting information

S1 File Detailed signal preprocessing and analysis. ECG, PPG, and BP preprocessing; beat segmentation and validation; DFA implementation; and statistical analysis [50–57].
(PDF)

S1 TableKruskal-Wallis tests across sleep stages within each group (DFA2).P-values are reported per signal and α exponent; *N* is the number of observations used.(PDF)

S2 Table Significant Bonferroni-adjusted pairwise Wilcoxon tests across stages within groups (DFA2). (PDF)

S3 Table Between-group (Healthy vs. Apnea) comparisons per stage (DFA2). Both Kruskal-Wallis (KW) and Wilcoxon p-values are shown; *N* denotes total observations.
(PDF)
