# Supplementary material for: Short- and long-term scaling behavior of blood pressure and pulse arrival time during sleep in healthy controls and patients with obstructive sleep apnea
Source: PLoS One. 2026 Jul 1;21(7):e0339755. doi: 10.1371/journal.pone.0339755 (PMC13322537; doi:10.1371/journal.pone.0339755)
Supplement: S1 File — ECG, PPG, and BP preprocessing; beat segmentation and validation; DFA implementation; and statistical analysis [50–57]. (PDF) [file pone.0339755.s001.pdf]

Supporting information

Detailed signal preprocessing

All analyses were performed in MATLAB (R2023a, The MathWorks Inc., Natick, MA, USA). For each subject, signals were truncated to the time span covered by the Portapres blood pressure (BP) recording to ensure temporal alignment across modalities. The following channels were analyzed in detail: electrocardiogram (ECG, $f_s = 200\,\mathrm{Hz}$), finger photoplethysmography (PPG, $f_s = 100\,\mathrm{Hz}$), and continuous finger BP from the Portapres system (Model 2, FMS, The Netherlands; volume-clamp method, $f_s = 200\,\mathrm{Hz}$). Where required, signals were resampled to a common time base using linear interpolation while preserving the original timing of R-peaks and pulse features.

ECG preprocessing and R-peak detection

ECG signals were first corrected for baseline wander. Baseline drift was estimated by applying a 0.25 s median filter to suppress QRS complexes, followed by a 0.75 s median filter to attenuate P and T waves. The resulting baseline estimate was then smoothed using a zero-phase, fifth-order Chebyshev Type II low-pass filter with a 4 Hz cutoff and subtracted from the original ECG. R-peaks were detected using the algorithm of Benítez et al. [30], and visually inspected for gross errors. The resulting R–R intervals (RRIs) served as the temporal reference for segmenting BP and PPG beats and for constructing the RRI time series used in DFA.

PPG preprocessing

PPG signals were preprocessed using a custom MATLAB routine designed to suppress high-frequency noise while preserving the physiological waveform. A fourth-order zero-phase Butterworth high-pass filter with a cutoff frequency of 10 Hz was applied to estimate the high-frequency noise component, which was then subtracted from the original signal. This procedure effectively reduced motion and sensor noise while maintaining the morphology of the pulsatile component.

BP preprocessing

For the Portapres BP signal, the same filtering procedure as for PPG was used, with the cutoff frequency of the high-pass filter set to 20 Hz and the sampling rate set to the native 200 Hz. This removed high-frequency artifacts while retaining the relevant physiological frequency range for systolic and diastolic pressure waveforms. In addition, calibration artifacts, finger-switching events, and brief signal dropouts were identified using the Portapres status flags and by visual inspection (see Fig. 2). Segments containing such artifacts were excluded from further analysis.

Beat segmentation, feature extraction, and validation

Beat-level segmentation and validation were performed to ensure morphological consistency across both BP and PPG signals.

BP beats and fiducial points

For the BP signal, individual beats were defined using consecutive ECG R-peaks as temporal boundaries. Each beat was extended by one full R–R interval beyond the subsequent R-peak to include the late diastolic phase. The extracted segments were linearly resampled to 150 points to obtain phase-aligned waveforms across cardiac cycles.

For each subject, an ideal reference waveform was computed as the mean of all resampled beats. Morphological similarity between each individual beat and the reference waveform was quantified using the Pearson correlation coefficient. Beats with similarity ≥ 0.8 were considered morphologically valid and retained for further analysis. Systolic (SBP) and diastolic (DBP) points were initially identified by mapping the maximum and minimum of the reference waveform onto each original beat and then refined within a ± 0.2 s window to ensure precise localization. Beats with SBP > 200 mmHg or DBP < 10 mmHg were excluded as physiologically implausible. The remaining beats were used to construct SBP and DBP time series.

PPG beats and fiducial points

PPG beats were segmented relative to ECG R-peaks to maintain synchrony between cardiac electrical activity and the peripheral pulse response. Consecutive R-peaks defined one complete PPG pulse cycle, and each segment was resampled to 200 points to standardize beat duration. For each subject, a reference PPG waveform was obtained by averaging all resampled beats. Morphological similarity between each beat and the reference was again quantified via the Pearson correlation coefficient; beats with similarity ≥ 0.6 were retained. Within each valid beat, the first local maximum after the onset was identified as the systolic PPG peak (PPG_{sys}), and the subsequent local minimum on the falling limb was identified as the diastolic PPG minimum (PPG_{dia}). Beats lacking a valid maximum–minimum pair or failing the similarity criterion were discarded. Segments affected by calibration, finger switching, or signal loss were also removed (Fig. 2).

Construction and validation of timing series

Pulse arrival times (PAT) were derived as described in the main Methods. Briefly, for Portapres BP signals:

- PAT_{sys}: interval from the ECG R-peak to the SBP maximum,
- PAT_{dia}: interval from the ECG R-peak to the diastolic BP minimum (DBP).

For PPG signals, PAT_{sys} and PAT_{dia} were defined analogously as the intervals from the R-peak to the PPG systolic peak and to the first diastolic PPG minimum following that peak, respectively. The following validation criteria were applied to the resulting beat-to-beat time series:

- **RRIs**: Interbeat intervals shorter than 0.33 s, longer than 2.0 s, 30% shorter than the previous RRI, or 60% longer than the previous RRI were discarded as non-normal beats, following previous work [17,31].
- **Systolic PATs**: For Portapres-derived PAT_{sys}, intervals shorter than 0.1 s, longer than 0.8 s, or 50% shorter/longer than the previous value were rejected. For PPG-derived PAT_{sys}, the corresponding time limits were extended to -0.3 s and 1.0 s, reflecting somewhat less reliable overall signal timing.
- **Diastolic PATs**: For both Portapres- and PPG-derived PAT_{dia}, intervals shorter than 0.3 s, longer than 3.0 s, or differing by more than $\pm 50\%$ from the previous value were excluded.
- **Blood pressure values**: BP values below 25 mmHg or above 250 mmHg were discarded as non-physiological.

After removing all invalid data points (beats), uninterrupted sequences with a minimum length of 20 consecutive measurements were retained for DFA.

DFA implementation

Detrended fluctuation analysis (DFA), originally introduced by Peng et al. [35] and later extended to higher-order polynomial detrending [20], was used to quantify correlations in noisy, nonstationary time series across multiple time scales s [36]. The integrated signal of length N is divided into non-overlapping segments of size s. Within each segment, a polynomial trend is removed, and the mean-square residuals are averaged to obtain the fluctuation function $F(s)$. For long-range correlated data, $F(s) \sim s^{\alpha}$, with scaling exponent $\alpha > 0.5$. This relation corresponds to a power-law in the power spectrum $P(f) \sim f^{-\beta}$, where $\beta = 2\alpha - 1$ [50]. In stationary cases ($\alpha < 1$), this is equivalent to an autocorrelation function scaling $C(s) \sim s^{-\gamma}$ with $\gamma = 1 - \beta$ [51]. DFA is preferred over conventional spectral or autocorrelation methods because of its robustness to trends, nonstationarities, and missing data [52–57]. In the present analysis, DFA with second-order polynomial detrending (DFA2) was applied to all nine time series (RRIs; Portapres-derived SBP, DBP, PAT_{sys}, PAT_{dia}; and PPG-derived PPG_{sys}, PPG_{dia}, PAT_{sys}, PAT_{dia}) separately for each subject and sleep stage. Fluctuation functions were computed over beat-based window sizes and averaged across uninterrupted episodes using statistical weights proportional to episode duration. Short-term scaling exponents α_1 were estimated over window sizes corresponding to 6–16 beats, and long-term exponents α_2 over 50–200 beats, as in previous work [17]. Only fits with coefficient of determination $r^2 > 0.9$ were retained. The resulting α_1 and α_2 values were then used in the group- and stage-level statistical analyses.

Statistical analysis

Normality of α_1 and α_2 distributions was assessed using the Shapiro-Wilk test; because most distributions deviated significantly from normality ($p < 0.05$), non-parametric methods were used throughout. Within each subject group (healthy, OSA), Kruskal-Wallis tests were applied across the five Portapres-derived measures (RRI, SBP, DBP, PAT_{sys}, PAT_{dia}) and the four corresponding PPG-derived measures for each sleep stage. Significant effects were followed by Bonferroni-corrected pairwise Wilcoxon tests to identify specific stage contrasts. Stage-related differences within each physiological measure were assessed using Kruskal-Wallis tests across wake, REM, N2, and N3, again followed by Bonferroni-corrected pairwise Wilcoxon tests where appropriate. Between-group differences (healthy vs. OSA) were evaluated separately for each physiological measure and sleep stage using Kruskal-Wallis and Wilcoxon tests. All analyses were performed in R (packages `rstatix` and `tidyverse`). The complete statistical results are provided in Tables S1–S3.
