# Supplementary material for: Short- and long-term scaling behavior of blood pressure and pulse arrival time during sleep in healthy controls and patients with obstructive sleep apnea
Source: PLoS One. 2026 Jul 1;21(7):e0339755. doi: 10.1371/journal.pone.0339755 (PMC13322537; doi:10.1371/journal.pone.0339755)
Supplement: S1 Table — P-values are reported per signal and α exponent; N is the number of observations used. (PDF) [file pone.0339755.s002.pdf]

Within-group stage effects

Table S1. Kruskal–Wallis tests across sleep stages within each group (DFA2). P-values are reported per signal and α exponent; N is the number of observations used.

α	Signal	Group	KW p	N
α_1	BP_{dia}	Apnoea	0.00669	72
α_1	BP_{dia}	Healthy	0.04450	92
α_1	PPG_{sys}	Apnoea	0.29600	72
α_1	PPG_{sys}	Healthy	4.00e−06	92
α_1	PPG_{dia}	Apnoea	0.03710	72
α_1	PPG_{dia}	Healthy	1.59e−05	92
α_1	PAT_{dia}	Apnoea	0.00261	72
α_1	PAT_{dia}	Healthy	0.13000	92
α_1	PPG-PAT_{sys}	Apnoea	0.12800	72
α_1	PPG-PAT_{sys}	Healthy	0.00538	92
α_1	PPG-PAT_{dia}	Apnoea	0.02230	72
α_1	PPG-PAT_{dia}	Healthy	0.00877	92
α_1	PAT_{sys}	Apnoea	0.80900	72
α_1	PAT_{sys}	Healthy	0.69300	92
α_1	BP_{sys}	Apnoea	0.04970	72
α_1	BP_{sys}	Healthy	0.00868	92
α_1	RRI	Apnoea	0.02260	72
α_1	RRI	Healthy	0.01480	92
α_2	BP_{dia}	Apnoea	0.13100	65
α_2	BP_{dia}	Healthy	5.72e−05	91
α_2	PPG_{sys}	Apnoea	1.41e−04	71
α_2	PPG_{sys}	Healthy	1.07e−04	91
α_2	PPG_{dia}	Apnoea	1.53e−05	71
α_2	PPG_{dia}	Healthy	2.89e−04	91
α_2	PAT_{dia}	Apnoea	9.86e−08	63
α_2	PAT_{dia}	Healthy	1.53e−08	89
α_2	PPG-PAT_{sys}	Apnoea	0.00209	68
α_2	PPG-PAT_{sys}	Healthy	7.76e−04	81
α_2	PPG-PAT_{dia}	Apnoea	1.98e−07	68
α_2	PPG-PAT_{dia}	Healthy	1.25e−05	89
α_2	PAT_{sys}	Apnoea	0.15900	63
α_2	PAT_{sys}	Healthy	0.00302	89
α_2	BP_{sys}	Apnoea	0.05500	65
α_2	BP_{sys}	Healthy	4.90e−07	92
α_2	RRI	Apnoea	1.27e−09	69
α_2	RRI	Healthy	4.07e−13	92
