# Supplementary material for: Short- and long-term scaling behavior of blood pressure and pulse arrival time during sleep in healthy controls and patients with obstructive sleep apnea
Source: PLoS One. 2026 Jul 1;21(7):e0339755. doi: 10.1371/journal.pone.0339755 (PMC13322537; doi:10.1371/journal.pone.0339755)
Supplement: S2 Table — (PDF) [file pone.0339755.s003.pdf]

Pairwise stage comparisons

Table S2. Significant Bonferroni-adjusted pairwise Wilcoxon tests across stages within groups (DFA2).

α	Signal	Group	Stage 1	Stage 2	n_1/n_2	p_{adj}
α_1	BP_{dia}	Apnoea	REM	N3	18/18	0.023
α_1	BP_{dia}	Healthy	wake	REM	23/23	0.034
α_1	PPG_{sys}	Healthy	wake	REM	23/23	1.72e−07
α_1	PPG_{sys}	Healthy	wake	N2	23/23	0.002
α_1	PPG_{sys}	Healthy	wake	N3	23/23	0.005
α_1	PPG_{dia}	Healthy	wake	REM	23/23	1.18e−05
α_1	PPG_{dia}	Healthy	REM	N2	23/23	0.019
α_1	PPG_{dia}	Healthy	REM	N3	23/23	0.019
α_1	PAT_{dia}	Apnoea	REM	N3	18/18	0.004
α_1	PPG-PAT_{sys}	Healthy	REM	N3	23/23	0.007
α_1	PPG-PAT_{dia}	Apnoea	REM	N3	18/18	0.049
α_1	PPG-PAT_{dia}	Healthy	REM	N3	23/23	0.014
α_1	BP_{sys}	Healthy	wake	REM	23/23	0.004
α_1	RRI	Apnoea	REM	N3	18/18	0.018
α_1	RRI	Healthy	REM	N3	23/23	0.019
α_2	BP_{dia}	Healthy	wake	N3	23/22	0.009
α_2	BP_{dia}	Healthy	REM	N2	23/23	0.005
α_2	BP_{dia}	Healthy	REM	N3	23/22	6.78e−04
α_2	PPG_{sys}	Apnoea	wake	N2	18/18	0.009
α_2	PPG_{sys}	Apnoea	wake	N3	18/17	0.009
α_2	PPG_{sys}	Apnoea	REM	N2	18/18	0.012
α_2	PPG_{sys}	Apnoea	REM	N3	18/17	0.010
α_2	PPG_{sys}	Healthy	wake	N3	23/22	0.014
α_2	PPG_{sys}	Healthy	REM	N2	23/23	0.011
α_2	PPG_{sys}	Healthy	REM	N3	23/22	2.62e−04
α_2	PPG_{dia}	Apnoea	wake	N2	18/18	0.002
α_2	PPG_{dia}	Apnoea	wake	N3	18/17	0.003
α_2	PPG_{dia}	Apnoea	REM	N2	18/18	0.006
α_2	PPG_{dia}	Apnoea	REM	N3	18/17	0.003
α_2	PPG_{dia}	Healthy	wake	N3	23/22	0.005
α_2	PPG_{dia}	Healthy	REM	N2	23/23	0.004
α_2	PPG_{dia}	Healthy	REM	N3	23/22	1.57e−04
α_2	PAT_{dia}	Apnoea	wake	N2	16/18	0.010
α_2	PAT_{dia}	Apnoea	wake	N3	16/14	0.009
α_2	PAT_{dia}	Apnoea	REM	N2	15/18	1.19e−05
α_2	PAT_{dia}	Apnoea	REM	N3	15/14	1.55e−07
α_2	PAT_{dia}	Healthy	wake	N3	21/22	5.22e−04
α_2	PAT_{dia}	Healthy	REM	N2	23/23	4.91e−05
α_2	PAT_{dia}	Healthy	REM	N3	23/22	1.62e−06
α_2	PPG-PAT_{sys}	Apnoea	wake	N3	18/15	0.036
α_2	PPG-PAT_{sys}	Apnoea	REM	N3	17/15	0.022
α_2	PPG-PAT_{sys}	Healthy	REM	N2	19/21	0.013
α_2	PPG-PAT_{sys}	Healthy	REM	N3	19/20	0.005
α_2	PPG-PAT_{dia}	Apnoea	wake	N2	18/18	0.002
α_2	PPG-PAT_{dia}	Apnoea	wake	N3	18/15	4.51e−04

(continued on next page)

(continued from previous page)

α	Signal	Group	Stage 1	Stage 2	n_1/n_2	p_{adj}
α_2	PPG-PAT$_{dia}$	Apnoea	REM	N2	17/18	2.06e−04
α_2	PPG-PAT$_{dia}$	Apnoea	REM	N3	17/15	1.19e−04
α_2	PPG-PAT$_{dia}$	Healthy	wake	N3	22/22	0.005
α_2	PPG-PAT$_{dia}$	Healthy	REM	N2	22/23	0.004
α_2	PPG-PAT$_{dia}$	Healthy	REM	N3	22/22	1.57e−04
α_2	PAT$_{sys}$	Healthy	wake	N3	21/22	0.017
α_2	PAT$_{sys}$	Healthy	REM	N3	23/22	0.011
α_2	BP$_{sys}$	Healthy	wake	N2	23/23	0.007
α_2	BP$_{sys}$	Healthy	wake	N3	23/23	9.18e−05
α_2	BP$_{sys}$	Healthy	REM	N2	23/23	0.002
α_2	BP$_{sys}$	Healthy	REM	N3	23/23	9.96e−05
α_2	RRI	Apnoea	wake	N2	18/18	1.34e−04
α_2	RRI	Apnoea	wake	N3	18/15	7.92e−05
α_2	RRI	Apnoea	REM	N2	18/18	4.40e−06
α_2	RRI	Apnoea	REM	N3	18/15	1.70e−05
α_2	RRI	Healthy	wake	N2	23/23	2.86e−07
α_2	RRI	Healthy	wake	N3	23/23	1.73e−07
α_2	RRI	Healthy	REM	N2	23/23	1.09e−06
α_2	RRI	Healthy	REM	N3	23/23	2.69e−07
