# Supplementary material for: Short- and long-term scaling behavior of blood pressure and pulse arrival time during sleep in healthy controls and patients with obstructive sleep apnea
Source: PLoS One. 2026 Jul 1;21(7):e0339755. doi: 10.1371/journal.pone.0339755 (PMC13322537; doi:10.1371/journal.pone.0339755)
Supplement: S3 Table — Both Kruskal-Wallis (KW) and Wilcoxon p-values are shown; N denotes total observations. (PDF) [file pone.0339755.s004.pdf]

Between-group differences by stage

Table S3. Between-group (Healthy vs. Apnea) comparisons per stage (DFA2). Both Kruskal-Wallis (KW) and Wilcoxon p-values are shown; N denotes total observations.

α	Signal	Stage	KW p	Wilcoxon p	N
α_1	BP_{dia}	wake	0.0519	0.0535	41
α_1	BP_{dia}	REM	0.1800	0.1850	41
α_1	BP_{dia}	N2	0.00654	0.00681	41
α_1	BP_{dia}	N3	0.00221	0.00231	41
α_1	PPG_{sys}	wake	0.1760	0.1800	41
α_1	PPG_{sys}	REM	0.00274	0.00287	41
α_1	PPG_{sys}	N2	0.00211	0.00221	41
α_1	PPG_{sys}	N3	0.00455	0.00474	41
α_1	PPG_{dia}	wake	0.0333	0.0344	41
α_1	PPG_{dia}	REM	0.00325	0.00339	41
α_1	PPG_{dia}	N2	4.76e−04	5.00e−04	41
α_1	PPG_{dia}	N3	0.00535	0.00558	41
α_1	PAT_{dia}	wake	0.2590	0.2640	41
α_1	PAT_{dia}	REM	0.2810	0.2870	41
α_1	PAT_{dia}	N2	0.0223	0.0231	41
α_1	PAT_{dia}	N3	0.0146	0.0151	41
α_1	$PPG\text{-}PAT_{sys}$	wake	0.00185	0.00193	41
α_1	$PPG\text{-}PAT_{sys}$	REM	0.00177	0.00185	41
α_1	$PPG\text{-}PAT_{sys}$	N2	4.10e−04	4.31e−04	41
α_1	$PPG\text{-}PAT_{sys}$	N3	7.01e−04	7.36e−04	41
α_1	$PPG\text{-}PAT_{dia}$	wake	0.1280	0.1310	41
α_1	$PPG\text{-}PAT_{dia}$	REM	0.0404	0.0417	41
α_1	$PPG\text{-}PAT_{dia}$	N2	0.5900	0.5990	41
α_1	$PPG\text{-}PAT_{dia}$	N3	0.8440	0.8540	41
α_1	PAT_{sys}	wake	0.0444	0.0458	41
α_1	PAT_{sys}	REM	0.0762	0.0784	41
α_1	PAT_{sys}	N2	0.0140	0.0145	41
α_1	PAT_{sys}	N3	0.0487	0.0503	41
α_1	BP_{sys}	wake	0.0168	0.0174	41
α_1	BP_{sys}	REM	0.0312	0.0322	41
α_1	BP_{sys}	N2	8.12e−05	8.58e−05	41
α_1	BP_{sys}	N3	7.72e−04	8.09e−04	41
α_1	RRI	wake	0.1030	0.1060	41
α_1	RRI	REM	0.0255	0.0264	41
α_1	RRI	N2	0.0146	0.0151	41
α_1	RRI	N3	0.0093	0.00966	41
α_2	BP_{dia}	wake	0.1150	0.1180	41
α_2	BP_{dia}	REM	0.1890	0.1940	38
α_2	BP_{dia}	N2	0.3650	0.3720	41
α_2	BP_{dia}	N3	0.6380	0.6500	36
α_2	PPG_{sys}	wake	0.4950	0.5030	41
α_2	PPG_{sys}	REM	0.7030	0.7130	41
α_2	PPG_{sys}	N2	0.0312	0.0323	41
α_2	PPG_{sys}	N3	0.4110	0.4200	39
α_2	PPG_{dia}	wake	0.0151	0.0156	41

(continued on next page)

(continued from previous page)

α	Signal	Stage	KW p	Wilcoxon p	N
α_2	PPG_{dia}	REM	0.2930	0.2990	41
α_2	PPG_{dia}	N2	0.5900	0.5990	41
α_2	PPG_{dia}	N3	0.9320	0.9440	39
α_2	PAT_{dia}	wake	0.8540	0.8660	37
α_2	PAT_{dia}	REM	0.4730	0.4830	38
α_2	PAT_{dia}	N2	0.2870	0.2930	41
α_2	PAT_{dia}	N3	0.3140	0.3270	36
α_2	$\mathrm{PPG\text{-}PAT}_{sys}$	wake	0.0807	0.0832	39
α_2	$\mathrm{PPG\text{-}PAT}_{sys}$	REM	0.6460	0.6570	36
α_2	$\mathrm{PPG\text{-}PAT}_{sys}$	N2	0.1670	0.1720	39
α_2	$\mathrm{PPG\text{-}PAT}_{sys}$	N3	0.3420	0.3510	35
α_2	$\mathrm{PPG\text{-}PAT}_{dia}$	wake	0.0472	0.0487	40
α_2	$\mathrm{PPG\text{-}PAT}_{dia}$	REM	0.0137	0.0143	39
α_2	$\mathrm{PPG\text{-}PAT}_{dia}$	N2	0.8640	0.8750	41
α_2	$\mathrm{PPG\text{-}PAT}_{dia}$	N3	0.8650	0.8770	37
α_2	PAT_{sys}	wake	0.0658	0.0681	37
α_2	PAT_{sys}	REM	0.1740	0.1810	38
α_2	PAT_{sys}	N2	0.0698	0.0719	41
α_2	PAT_{sys}	N3	0.5810	0.5970	36
α_2	BP_{sys}	wake	0.00629	0.00655	41
α_2	BP_{sys}	REM	0.41100	0.42000	38
α_2	BP_{sys}	N2	0.32500	0.33100	41
α_2	BP_{sys}	N3	0.43400	0.44300	37
α_2	RRI	wake	0.18000	0.18500	41
α_2	RRI	REM	0.27600	0.28100	41
α_2	RRI	N2	0.47800	0.48600	41
α_2	RRI	N3	0.95200	0.96400	38
